# Organisation and Control

**Published:** 1914-08-29

**Authors:** 


### A STATE OF WAR.

### The Country and the Care of the Sick and Wounded.

### ORGANISATION AND CONTROL.

A great deal of avoidable trouble, disappointment, and difficulty will be prevented if the multitude of good people who wish to do something at this time to help practically in the cause of the sick and wounded can be brought to understand some essential points in the organisation and control of these matters. First, then, it should be clearly understood that the British Red Cross Society has many duties and responsibilities, but so far as the provision for the sick and wounded is concerned, the latest arrangements make this Society the sieve upon which the Admiralty and the War Office rely, initially, in regard to the various offers of accommodation for hospital and semi-convalescent patients, and for service on the part of matrons and nurses and other helpers who are anxious to take some part. All offers of buildings, hospitals, private houses, and so forth are handed to the British Red Cross Society, which codifies and arranges them by the counties in which they are situated, with due regard to the relation they may bear to the various military hospitals, because these relations may influence the manner in which each offer, when approved, may be employed in the future. All offers from public institutions have been placed on a special list, and those fully staffed and equipped have been particularly noted as likely to be useful.

### Voluntary Aid Detachment Hospitals.

In the case of temporary hospitals to be established by Voluntary Aid Detachments, these form an integral and important link in the Territorial military organisation. No Voluntary Aid Detachment will, it is understood, be utilised except in the district in which it is registered, although the military authorities may ask for volunteers for duty elsewhere from specially selected detachments. At present Voluntary Aid Detachments are asked not to prepare any actual hospitals, but to have schemes for them ready to open at any moment, when required by the military authorities. This class of hospitals belongs to the category of temporary hospitals, and their equipment, except in the case of a small supply of drugs and dressings, should not necessarily consist of articles except such as can be obtained from neighbouring houses.

### Some Important Regulations.

In regard to auxiliary home hospitals, the British Red Cross Society desires to impress upon everybody the importance of those concerned precisely adhering to the following regulations:—

(1) That no hospital should be actually prepared before the mobilisation order of that hospital has been received from the military authority, in order that *personnel* and equipment which may be urgently needed elsewhere shall not be uselessly locked up.

(2) That funds should not be prematurely laid out on the preparation of these hospitals which might be more usefully expended in other directions.

(3) That the work of institutions intended for other purposes, such as schools and colleges, must not be interfered with, except on military requisition or in consultation with the educational authorities.

(4) That Voluntary Aid Detachments have no authority whatever to take over of themselves any public or private building.

It is particularly desired that gentlemen and ladies offering assistance in the shape of private houses will husband their resources, and remain ready to put their carefully prepared plans into execution when invited to do so.

### Where Responsibility is Vested.

The British Red Cross Society is, we understand, employing the services of retired members of the R.A.M.C. to inspect some of the more important buildings and houses offered, which seem initially to be sufficiently important and *prima facie* suitable. The British Red Cross Society undertakes no responsibility for the equipment or the hygienic or structural quality of the accommodation provided for the sick and wounded, nor in regard to the medical, nursing, commissariat, and executive work connected with any hospital or other provision. All these matters are undertaken entirely by the departments of the Medical Directors-General of the Navy and Army respectively, and it seems important to make this position clear, because no hospital or semi-convalescent institution of any kind can be utilised for the reception of sick and wounded sailors and soldiers, unless the Admiralty and War Office authorities have approved its equipment, staff, and administrative arrangements.

We believe a knowledge of these facts will give confidence to the friends of our sick and wounded, and to the public also. It would be a terrible calamity to bring sick or wounded men from the scene of conflict into some hospital or other building where the arrangements were not of the very best from the surgical, hygienic, and administrative point of view. Economical considerations alone, in the case of a war which, like the present gigantic conflict between European nations, seems likely to be indefinitely prolonged, render it essential that the ambulance arrangements, and every detail of hospital and convalescent provision for the care, treatment, and speedy recovery of the sick and wounded, shall be excellent. It is essential to afford the most perfect guarantees that, with God's blessing, every patient will have the maximum of facility for his speedy restoration to health.

